# Role of oxygen and the OxyR protein in the response to iron limitation in *Rhodobacter sphaeroides*

**DOI:** 10.1186/1471-2164-15-794

**Published:** 2014-09-15

**Authors:** Bernhard Remes, Bork A Berghoff, Konrad U Förstner, Gabriele Klug

**Affiliations:** Institut für Mikrobiologie und Molekularbiologie, IFZ, Justus-Liebig-Universität, Heinrich-Buff-Ring 26, 35392 Giessen, Germany; Department of Cell and Molecular Biology, Biomedical Centre, Uppsala University, Box 596, 75124 Uppsala, Sweden; Institutes for Molecular Infection Biology, University of Würzburg, 97080 Würzburg, Germany; Research Centre for Infectious Diseases, University of Würzburg, 97080 Würzburg, Germany

**Keywords:** *Rhodobacter sphaeroides*, Transcriptomics, Iron limitation, OxyR, RNAseq, Oxidative stress

## Abstract

**Background:**

High intracellular levels of unbound iron can contribute to the production of reactive oxygen species (ROS) via the Fenton reaction, while depletion of iron limits the availability of iron-containing proteins, some of which have important functions in defence against oxidative stress. Vice versa increased ROS levels lead to the damage of proteins with iron sulphur centres. Thus, organisms have to coordinate and balance their responses to oxidative stress and iron availability. Our knowledge of the molecular mechanisms underlying the co-regulation of these responses remains limited. To discriminate between a direct cellular response to iron limitation and indirect responses, which are the consequence of increased levels of ROS, we compared the response of the α-proteobacterium *Rhodobacter sphaeroides* to iron limitation in the presence or absence of oxygen.

**Results:**

One third of all genes with altered expression under iron limitation showed a response that was independent of oxygen availability. The other iron-regulated genes showed different responses in oxic or anoxic conditions and were grouped into six clusters based on the different expression profiles. For two of these clusters, induction in response to iron limitation under oxic conditions was dependent on the OxyR regulatory protein. An OxyR mutant showed increased ROS production and impaired growth under iron limitation.

**Conclusion:**

Some *R. sphaeroides* genes respond to iron limitation irrespective of oxygen availability. These genes therefore reflect a “core iron response” that is independent of potential ROS production under oxic, iron-limiting conditions. However, the regulation of most of the iron-responsive genes was biased by oxygen availability. Most strikingly, the OxyR-dependent activation of a subset of genes upon iron limitation under oxic conditions, including many genes with a role in iron metabolism, revealed that elevated ROS levels were an important trigger for this response. OxyR thus provides a regulatory link between the responses to oxidative stress and to iron limitation in *R. sphaeroides*.

**Electronic supplementary material:**

The online version of this article (doi:10.1186/1471-2164-15-794) contains supplementary material, which is available to authorized users.

## Background

All aerobic bacteria produce toxic oxygen derivatives as by-products of their metabolism. Therefore, they have evolved complex defence and repair mechanisms to protect themselves from the damaging effects of emerging reactive oxygen species (ROS) [1]. These mechanisms allow bacteria to cope with oxidative stress and the imbalance between ROS-generating and ROS-defence processes [2, 3]. A direct detoxification of ROS is catalysed by such enzymes as superoxide dismutases (SOD), catalases and peroxidases [4, 5]. Surprisingly, strictly anaerobic bacteria express comparable detoxifying systems, such as the SOD, but little is known about their role and the possibility of ROS protection in these organisms [1, 6].

As a cofactor of several enzymes and regulatory proteins, iron is an essential element for living organisms. Iron is mainly present in two forms, either as soluble ferrous iron Fe(II) or as insoluble ferric iron Fe(III). In oxic environments, only Fe(III) is energetically stable [7], while in anoxic habitats, reduction to Fe(II) occurs chemically by organic compounds, such as sulphides [8]. Bacteria possess numerous highly efficient iron acquisition systems to scavenge iron from the environment under iron-restricted conditions, such as the synthesis and secretion of high affinity extracellular ferric chelators called siderophores [9, 10]. However, siderophores can have physiological roles aside from those involved in iron acquisition; one such example is siderophores acting as protectors against oxidative stress [11]. Although iron is essential for bacteria, iron potentiates oxygen toxicity by the production of hydroxyl radicals via the Fenton reaction. Fenton-like reactions are not restricted to iron as the reactive metal component. Other metals, such as copper, which is required for many cellular enzymes, such as cytochrome oxidase or SOD [12, 13], also catalyse the generation of reactive hydroxyl radicals that cause cellular damage [14]. On the other hand, iron is required for some enzymes involved in ROS detoxification (e.g., catalases and Fe-containing SOD) or in sensing oxidative stress (e.g., SoxRS). Thus, iron limitation interferes with the oxidative stress response. Iron limitation in photosynthetic organisms, such as *Anabaena* sp. Strain PCC 7120, *Synechocystis* sp. Strain PCC 6803 or *R. sphaeroides*, resulted in a 2- to 10-fold increase in ROS levels compared with those found in cells grown with appropriate iron supplementation [15, 16]. However, when non-photosynthetic *Escherichia coli* or *Bacillus subtilis* cells were iron starved, they did not exhibit a significant increase in the ROS levels [15]. Therefore, Latifi et al. proposed in 2005 that oxidative damage induced by iron starvation could be a characteristic of photosynthetic organisms. However, life in the presence of oxygen requires a strict regulation of iron metabolism in all organisms.

The oxidative stress response in *E. coli* mainly relies on the genes of the OxyR and SoxRS regulons [3]. A *Salmonella oxyR* deletion mutant was discovered to be hypersensitive to hydrogen peroxide (H_2_O_2_) [17]. Furthermore, the OxyR protein positively regulates the expression of the ferric uptake regulator *fur* gene in response to H_2_O_2_ [9]. In *E. coli* and in many other bacteria, the Fur protein is the main regulator of iron-dependent gene expression (reviewed in e.g., [18]). This pattern of gene expression constitutes a regulatory link between oxidative stress responses and iron homeostasis. Due to increased Fur levels during H_2_O_2_ stress, Fe(II) binding and iron storage is induced, leading to reduced free iron levels, which in turn help bacteria to cope with oxidative stress [9].

Experimental data and bioinformatic analyses suggest that in α-proteobacteria, iron regulation mainly occurs via regulators other than Fur [19], and nothing is known regarding the regulatory link between iron metabolism and defence against oxidative stress. *R. sphaeroides* is a facultative photosynthetic bacterium, which performs aerobic respiration in the presence of oxygen. In anoxic conditions in the light, anoxygenic photosynthesis generates ATP, while in the dark and in the presence of an electron acceptor, such as dimethyl sulphoxide (DMSO), anaerobic respiration can be performed. An earlier transcriptome study revealed that many of the genes involved in iron metabolism are induced in response to H_2_O_2_, demonstrating a strong correlation between oxidative stress responses and iron metabolism in this bacterium [20]. Several of these regulated genes are controlled by the intensively studied OxyR regulator [21]. Transcriptome studies also identified genes affected by iron limitation in *R. sphaeroides* and revealed that the Fur-related proteins Fur/Mur and Irr have no major function in activating gene expression in response to iron limitation [16]. The goal of this study is to discriminate between direct effects of iron limitation on gene expression and indirect effects, which are caused by the oxidative stress that occurs due to iron depletion [22]. Towards this end, the effects of iron limitation on global gene expression in anoxic conditions and oxic conditions were compared. Although there was a strong correlation between the two data sets, a number of genes exclusively responded to iron-limiting conditions in the presence of oxygen, while others responded only under anoxic conditions. Furthermore, we provide evidence for an important role of OxyR in iron-dependent activation of genes under oxic growth conditions.

## Results and discussion

### A subset of genes responds to iron limitation independently of oxygen availability

Only few global gene expression studies of iron limitation performed in oxic conditions simultaneously examined gene expression under iron-limiting, anoxic conditions (e.g., [23]). In order to discriminate the effects on gene expression that are a direct consequence of iron limitation from those caused by increased ROS levels due to iron limitation [22], we analysed *R. sphaeroides* transcriptomes in both oxic (25–30 μM oxygen, low oxygen tension) and anoxic conditions (using DMSO as terminal electron acceptor). To generate iron limitation, cultures were grown without external iron in the presence of the iron chelator 2,2′-dipyridyl (30 μM). The high metabolic versatility of *R. sphaeroides* allows such comparative studies, as it is capable to grow by both aerobic respiration and anaerobic respiration (if an alternative electron acceptor is available).

Total RNA from three independent control cultures (iron-replete conditions) and three independent iron-limiting cultures was isolated, pooled and used for RNAseq analysis. We normalised the data using READemption [24] with default parameters using segemehl version 0.1.3 [25] for the read alignment (accuracy parameter set to 95%). Further differential gene expression analysis was performed with DESeq 1.12.0 [26] only considering genes that show in at least one of the conditions a RPKM [27] value of ≥ 5.0. Relative changes in RNA levels from iron-depleted cells compared with control cells were calculated and considered to be regulated in case of a log_2_ fold change of > 1 or < -1 (Additional file 1: Table S1). Since only one sequencing run was performed, statistical evaluation of this dataset was limited. We therefore validated our results by Microarray analyses. For this approach the RNA of three independent experiments of control and iron-limiting cultures was pooled and hybridised to one array. Transcriptome profiles were analysed on two arrays including six biological replicates and confirmed the changes observed by RNAseq, for most genes (Additional file 1: Table S1). However, for most genes, fold changes as determined by RNAseq were distinctly higher than those determined by microarray analysis due to the lower sensitivity of the latter method. For several strongly regulated genes, a quantification of transcript levels was performed by real-time RT-PCR and confirmed the higher fold changes determined by RNAseq (Table 1). Therefore, we mainly referred to the RNAseq data for our comparative analysis.

**Table 1:** **Quantified log**_**2**_ **fold changes in response to iron-limitation**

Cluster		Oxic conditions		Anoxic conditions	
Gene	Description	2.4.1 WT	2.4.1Δ***oxyR***	2.4.1 WT	2.4.1Δ***oxyR***
Cluster I					
RSP_0069	*fliC*, flagellar protein	-2.89	-2.33	-1.17	-1.05
RSP_0850	*mbfA,* ferritin-like protein	-1.23	-1.09	-1.53	-1.01
RSP_2395	*ccpA,* cytochrome c peroxidase	0.01	0.46	-0.81	-0.33
RSP_6158	*puc2A*, light-harvesting complex	-5.63	-6.33	-1.01	-1.89
Cluster II					
RSP_0261	*bchY*, photopigment biosynthesis	-1.44	-2.21	-0.66	-0. 22
RSP_0285	*bchN*, photopigment biosynthesis	-5.88	-4.84	-0.32	-0.63
RSP_0288	*bchL*, photopigment biosynthesis	-4.12	-5.06	-0.44	-0.78
RSP_0679	*hemC*, Vitamin B12 synthesis	-1.15	-1.50	0.16	-0.28
RSP_1565	*appA*, blue light sensor	-1.85	-1.12	-0.82	-0.45
Cluster III					
RSP_0920	*exbB,* biopolymer transport protein	5.94	0.10***	3.03	3.02
RSP_0921	*exbD,* biopolymer transport protein	4.38	0.96**	2.28	2.11
RSP_0922	*tonB*, iron transporter	1.83	0.30**	1.63	1.34
RSP_1438	ABC ferric transporter	3.02	-0.46**	2.47	2.15
RSP_1547	*bfd*, bacterioferritin associated ferredoxin	3.21	0.91*	2.22	1.95
RSP_1548	*irpA,* iron-regulated protein	5.40	0.78**	3.67	2.57
RSP_2913	*afuA*, ABC siderophore transporter	6.08	2.02**	2.25	2.03
RSP_6006	*hemP,* hemin uptake protein	5.51	2.16***	2.73	2.66
Cluster IV					
RSP_0130	*metI*, methionine uptake transporter	3.07	3.84	2.34	1.78
RSP_1109	*cysK*, cysteine synthase	2.23	3.07	3.23	3.27
RSP_1818	*feoA1,* ferrous iron transport protein	-1.12	-1.21	0.58	0.51
RSP_3323	putative flavoprotein	1.90	1.56	2.84	2.64
RSP_3696	*cysA*, ABC sulfate transporter	3.96	3.65	2.23	1.03*
RSP_3697	*cysP*, ABC sulfate transporter	4.01	3.34	3.39	3.68
RSP_6020	*feoA2,* ferrous iron transport protein	0.04	-0.17	1.37	1.18
Cluster V					
RSP_0994	*phaD*, NADH dehydrogenase	0.29	-0.46	-1.61	-1.35
RSP_2311	*groEL,* chaperonin	1.49	-1.06**	1.80	1.61
RSP_3567	*znuB*, ABC zinc transporter	0.08	0.03	-0.57	-0.13
Cluster VI					
RSP_0434	*sufD*, iron-regulated ABC transporter	3.40	-1.47***	0.40	0.69
RSP_0437	*sufC,* iron-regulated ABC transporter	1.75	-0.83***	-1.35	-0.66
RSP_0439	hypothetical protein	1.49	-0.76**	-0.72	-0.38
RSP_0440	*sufB*, iron-regulated ABC transporter	2.60	-1.04*	0.01	0.44
RSP_0443	*iscR,* iron sulfur cluster regulator	2.45	0.28*	-0.76	0.47
RSP_0906	*sitC, ABC* Mn^2+^ transporter	2.91	0.95*	0.50	-0.35
RSP_3568	*znuC,* ABC zinc transporter	1.22	-0.71**	0.29	0.92
Other genes					
RSP_0601	*rpoH*_*II*_, RNA polymerase sigma factor	1.52	-1.16***	0.48	0.88
RSP_1092	*rpoE*, sigma factor	0.84	-1.58***	0.75	0.59
RSP_2779	*katE*, catalase	1.19	-1.13*	0.88	1.38

Real-time RT-PCR was used to investigate the relative expression of strongly regulated genes in oxic or anoxic conditions. Values are normalised to *rpoZ* and to the control under normal iron conditions. The data represent the mean of at least three independent experiments. A p-value was computed using the student’s *t* test. Variations were considered statistically significant when the p-value was ≤0.05.

*significant at p ≤0.05; **significant at p ≤0.01; ***significant at p ≤0.001.

**Figure 1:**
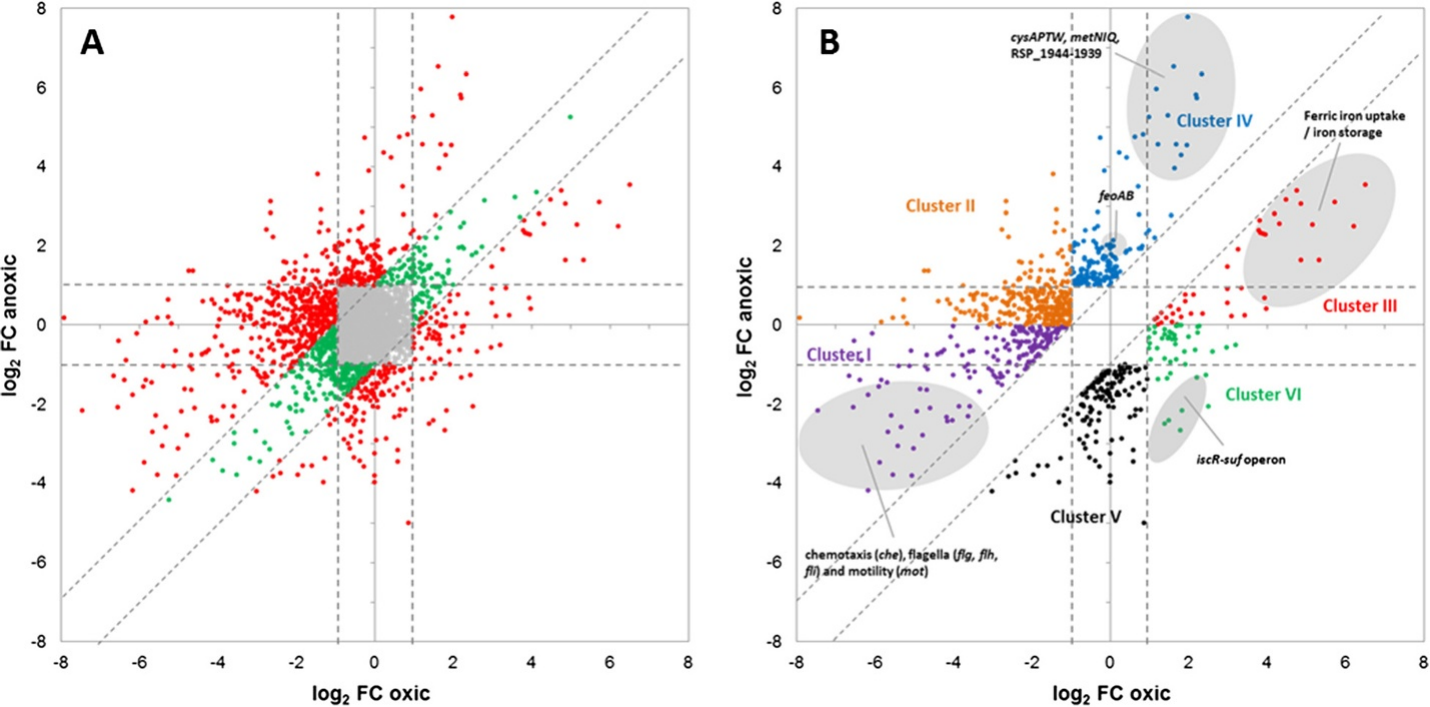
**Correlation between oxic and anoxic RNAseq analysis.** The scatter-plot represents a comparison of log_2_ fold changes between the oxic and anoxic RNAseq data sets. **(A)** Colour is used for the regulated genes (log_2_ > 1 or < -1, green and red spots, respectively) to indicate whether changes are of similar magnitude under both conditions (log_2_ ratio difference between approaches < 1, green spots) or are biased towards one condition (log_2_ ratio difference > 1, red spots). Non-regulated genes (log_2_ < 1 and > -1) are shown as grey spots. **(B)** Genes were grouped into six clusters according to their expression pattern as described in Results. For a complete list of genes and information on their functions, see Additional file 1: Table S1.

Figure 1 displays the comparison of log_2_ ratios between the iron-depleted and control cultures under oxic and anoxic conditions from the RNAseq datasets for all of the 3657 protein-coding genes that show in at least one of the conditions a RPKM [27] value of > =5.0. We considered those genes showing a fold change in response to iron regulation of log_2_ < 1 and > -1 as not being regulated by iron. These 2339 non-iron-regulated genes are represented by the grey spots in the middle square of Figure 1A. We considered the 1318 genes showing expression changes of log_2_ < 1 or > -1 as being regulated by iron. Of these 1357 iron-regulated genes, 373 genes, of which 270 were repressed and 103 were induced, showed similar responses to iron limitation independent of oxygen availability (the difference between the log_2_ ratios under oxic and anoxic conditions was < 1). These genes are represented by green spots (Additional file 2: Table S2). The oxygen-independent iron-regulated genes have diverse functions: most of the genes encode hypothetical proteins, transcriptional regulators or ABC transporters; several genes function in motility and chemotaxis, encode ribosomal proteins or components of dehydrogenases. Six of these genes are predicted to have functions related to iron metabolism: *bfr* encodes a bacterioferritin; RSP_3056 and RSP_4273 encode TonB-dependent iron siderophore receptors; and RSP_3391, RSP_3414 and RSP_4271 encode the ABC Fe siderophore transporter. This expression pattern indicates that these genes directly respond to iron limitation and not to a change in ROS levels. In previous studies, we showed that only 5 of these 373 genes responded to ^1^O_2_ stress conditions, whereas 20 of the genes responded to H_2_O_2_ stress conditions [20, 28]. Furthermore, 35 of the genes were expressed in an Irr-dependent manner, while 10 of the genes showed an opposite expression pattern in a strain lacking the Fur ortholog Fur/Mur (highlighted in Additional file 2: Table S2) [16, 22] *.* Thus, the previously studied iron regulators of alpha-proteobacteria Fur/Mur and Irr have no major involvement in the ROS-independent regulation of iron-responsive genes. The regulators involved in this “core iron response” still await identification.

### Genes with oxygen-dependent responses to iron limitation show different distinct expression patterns

For the majority of the 1318 iron-regulated protein-coding genes (945 genes, red spots in Figure 1A), the response to iron was dependent on oxygen availability. Because these genes showed distinct expression patterns, we assigned them to different clusters for further analysis and discussion. Figure 1B depicts these different clusters of genes, omitting all genes that are not regulated by iron or that show an oxygen-independent response to iron limitation (grey and green spots in Figure 1A).

One hundred eighty-four genes with a stronger down-regulation in response to iron limitation in oxic conditions than in anoxic conditions are grouped in cluster I (Figure 1B, purple spots). Most of these genes have a function in chemotaxis and motility (*che, mot, flg, flh, fli*). It was shown previously that these genes are also down-regulated in response to oxidative stress in *R. sphaeroides* [20], indicating that these genes are likely repressed by ROS in particular. An effect of iron on the expression of motility genes was also observed in *Helicobacter pylori* [29], *Vibrio cholerae* [30] and *Pseudomonas fluorescens* [31]. Cluster I includes also most genes for the synthesis of pigment-binding proteins *(pufBALMX*, *pucAB*, *puc2BA*). Several genes of the *nuo* operon encoding subunits of the NADH dehydrogenase complex are also part of cluster I. This enzymatic complex comprises iron sulphur clusters. Repression under oxic conditions when the amount of iron cofactor is limited therefore reduces the production of these proteins and may also reduce the production of further ROS. Under anoxic conditions, the expression of *nuo* genes is very low, and no additional repression by iron limitation is necessary. The RNAseq data for these experiments are publicly available through the NCBI’s GEO database (accession number GSE47182).

Cluster II (Figure 1B, orange spots) consists of 330 genes that show an expression change in response to iron limitation of log_2_ < -1 in oxic conditions and log_2_ > 0 in anoxic conditions. Most of these genes function in the synthesis of bacteriochlorophyll (e.g., *bchE, bchC, bchJ, bchY),* the synthesis of heme and bacteriochlorophyll precursors *(hemN, hemC, hemZ, hemB, hemA*), or the synthesis of carotenoids (*crtF, crtE, crtC, crtA*). Because bacteriochlorophyll synthesis and energy conversion in the reaction centre require iron [32, 33], a repression of genes that function in photosynthesis in response to iron limitation is meaningful. However, the exclusive repression under oxic conditions indicates that the repression is a response to the elevated ROS levels that occur due to iron limitation. This result is in agreement with the repressing effect of oxidative stress and photooxidative stress on these genes [20, 28].

Cluster III includes 51 genes (Figure 1B, red spots) that exhibit stronger upregulation in response to iron limitation in oxic conditions than in anoxic conditions. Many genes with predicted functions in ferric iron uptake and storage are among this cluster, including *exbBD* and *tonB*, RSP_1438-1440 encoding a ferrichrome transporter, *bfd* encoding a bacterioferritin, *irpA* encoding an iron-regulated protein, genes encoding Fe^3+^-siderophore transporters and *hemP* encoding an iron uptake protein. An induction of genes for ferric iron uptake and transport under iron-limitation conditions helps to counteract the iron limitation. Under anoxic conditions, iron is mostly present as ferrous iron, and a strong upregulation of these genes would not be appropriate.

Cluster IV comprises 160 genes (Figure 1B, blue spots) that are exclusively or much more strongly upregulated in anoxic iron-limiting conditions. Among these genes, we found the ferrous transport system *feoAB*, several genes involved in cysteine and sulphur metabolism, including RSP_1944-1939 (sulphate reduction) and *cysK*, *cysAPTW*, and *metNIQ* (methionine uptake) and genes with other functions, such as RSP_3323 encoding a flavoprotein, RSP_2890-2891 encoding a copper transport protein and CueR, a regulator of RSP_2890-2891 [34]. An upregulation of the ferrous transport system *feoAB* and other metal transport systems would help to counteract iron limitation under anoxic conditions.

Cluster V includes 164 genes (Figure 1B, black spots) with an exclusive or much stronger repression in response to iron limitation under anoxic conditions compared with oxic conditions, and these genes have very diverse functions. Two of the genes, *groES* and *groEL*, have a role in the heat shock response, 13 of the genes encode ribosomal proteins, 20 of the genes encode transporters and 4 of the genes encode putative transcriptional regulators. In addition, genes for hydrogenase production and regulation (*hup*, *hyp*) fall into this cluster. The iron-containing hydrogenases might be non-essential, and their down-regulation upon iron limitation would therefore free up trace amounts of iron for more crucial proteins. Genes for hydrogenase production and regulation are only weakly expressed in oxic conditions, and no further repression under iron-limitation conditions is required (GEO series GSE47182).

Remarkably, 56 genes (Figure 1B, green spots) grouped in cluster VI showed opposite responses to iron limitation in the presence or in the absence of oxygen. Under iron depletion, most genes of the *isc-suf* operon for iron-sulphur cluster assembly were induced in an oxic environment but repressed under anoxic conditions. An increase of the Suf machinery to enable iron-sulphur cluster assembly under low iron concentrations is understandable. The demand for repair of damaged iron-sulphur clusters is higher under oxic conditions, as elevated ROS levels destabilise Fe-S clusters. Hence, it is not surprising that these genes were also upregulated in the presence of H_2_O_2_ stress [20, 28]. In anoxic conditions, Fe-S clusters remain stable; consequently, the *isc-suf* operon is repressed. However, several metal transporters, such as *modA* and *modD* for molybdate transport or RSP_2890-2891 for copper transport, are exclusively induced in anoxic conditions, while genes coding for sulphate/thiosulphate transporters (*cysAPTW*) or methionine uptake transporters (*metNIQ*) are more strongly induced compared with oxic conditions. We presume that in iron-limiting anoxic conditions, a change in the demand for cofactors takes place and iron is partly replaced by other metals.

### Role of the OxyR protein in the response of *R. sphaeroides*to iron limitation in oxic and anoxic conditions

A response of many iron homeostasis genes, including *sufBCD*, *bfd-bfr* or *exbBD*-*tonB*, to H_2_O_2_ exposure in *R. sphaeroides* was previously observed, and an influence of the OxyR protein was revealed for most of these genes [20, 21, 28]. To elucidate the role of OxyR in iron-starved cells, we compared log_2_ ratios in response to iron limitation using real-time RT-PCR for the wild type strain and a strain lacking OxyR (2.4.1Δ*oxyR*) due to a chromosomal deletion [35]. We selected those genes that showed a strong regulation in the RNAseq or microarray analyses (Additional file 1: Table S1). Genes from all six clusters were selected to test for a potential cluster specificity of OxyR.

The stronger induction in response to iron starvation in oxic conditions of cluster III genes, namely *exbBD, tonB, bfd, irpA*, *afuA*, *hemP* and RSP_1438 (ABC ferric transporter), revealed by RNAseq analysis was confirmed by real-time RT-PCR (Table 1). However, the induction in oxic conditions was significantly reduced in the *oxyR* deletion strain, while the *oxyR* deletion had no significant effect under anoxic conditions. A similar expression pattern was observed for cluster VI genes, represented by genes of the *isc-suf* operon, the *sitABCD* operon (ABC Mn^2+^/Fe^2+^ transport system [36]) and *znuC* (ABC zinc transporter) (Table 1). Induction of these genes in response to iron limitation under oxic conditions was significantly dependent on OxyR.

OxyR-dependent upregulation of cluster III and cluster VI genes may prevent a stronger oxidative stress; e.g., by removing free iron (bacterioferritin), removing oxygen (cytochrome c oxidase) or upregulating the oxidative stress response (RpoE). Our data provide the first clue regarding a regulatory link between iron metabolism and oxidative stress defence in an alpha-proteobacterium: OxyR, which is activated by oxidative stress and consequently affects the expression of genes involved in the oxidative stress response, also activates genes important for iron metabolism in oxic conditions with iron limitation.

The fact that most cluster III genes showed some activation under oxic iron-limiting conditions in the *oxyR* mutant implies that factors other than OxyR are responsible for maximal induction. These factors may also trigger the observed activation of cluster III genes under anoxic conditions, which was independent of OxyR.

While all of the tested genes for ferric iron transporter systems (cluster III genes) were regulated in an OxyR-dependent manner, the ferrous transport system *feoAB* and other cluster IV genes were not influenced by OxyR (Table 1). The same result holds true for the remaining clusters I, II and V. The only exception was the *groEL* operon (cluster V); however, the cluster V expression pattern was not confirmed by real-time RT-PCR analysis.

The possibility that the expression discrepancies in the background of ∆*oxyR* between oxic and anoxic conditions were DMSO dependent can be excluded. We compared transcript level of wild type and ∆*oxyR* cultures grown in oxic conditions in presence or absence of DMSO. No significant changes could be observed due to DMSO addition (p-value ≤0.05) (Additional file 3: Figure S1).

In a previous study, we reported that a consensus sequence for the DNA binding site of OxyR does not exist in *Rhodobacter* [21]. Only the pattern TN_11_A, defining the minimum binding site for LysR-type regulators, was present in the majority of OxyR binding regions. In an effort to obtain more detailed information about the promoter specificity of the genes in the six clusters, we used the MEME program (http://meme.sdsc.edu./meme/) to identify conserved, overrepresented DNA motifs in the upstream promoter region of the selected genes [37]. As the search did not reveal clear DNA motifs for an individual cluster, we assume that several transcription factors are responsible for the differential expression or that the consensus for the binding sequence is very weak, as in the case of OxyR.

### Iron starvation impairs growth of strain 2.4.1*oxyR*in oxic, but not in anoxic, conditions

**Figure 2:**
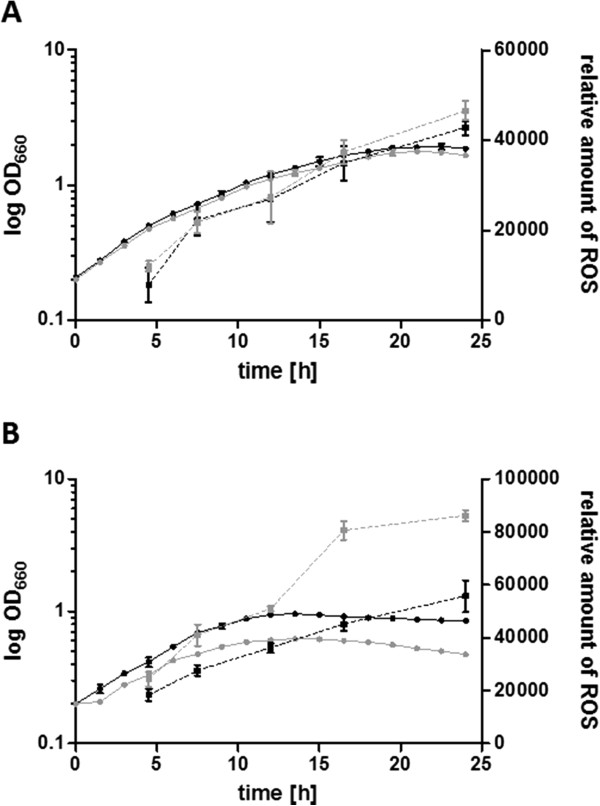
**Growth curves and ROS level measurements.** Characterisation of wild type *R. sphaeroides* (black) and the 2.4.1∆*oxyR* mutant (grey) was performed in oxic conditions in the presence **(A)** or absence **(B)** of iron. The optical density at 660 nm (OD_660_) was determined over time, and growth is indicated as continuous line. The intracellular levels of ROS (squares) are presented in arbitrary units. Both data sets represent the mean of at least three independent experiments, and the error bars indicate the standard deviation.

Based on the findings that many genes with a role in iron homeostasis were expressed in an OxyR-dependent manner only in oxic conditions, we assumed that iron deprivation along with oxic conditions would lead to impaired growth of an *oxyR* deletion strain. A growth defect under iron limitation was previously observed for a *Pseudomonas aeruginosa* PAO1 strain, which lacks OxyR [38]. We therefore monitored the growth of the *oxyR* deletion strain 2.4.1*oxyR* under normal growth conditions and under iron starvation in oxic or anoxic conditions and compared it with that of the parental wild type strain. During the exponential phase in oxic conditions, the wild type and *oxyR* deletion mutant showed similar doubling times, regardless of iron availability (Table 2). In the presence of iron, both strains reached a comparable end-OD (Figure 2A). However, under iron limitation in oxic conditions, the mutant showed a significantly lower end-OD (Figure 2B), indicating an important role of OxyR under these conditions.

**Table 2:** **Doubling times of wild type and 2.4.1∆** ***oxyR***

	Doubling times (h)	
Strain	Oxic	Anoxic
Wild type	3.9 ± 0.2	12.5 ± 1.2
Wild type -Fe	4.3 ± 0.3	16.1 ± 1.5
2.4.1Δ*oxyR*	4.2 ± 0.2	22.4 ± 2.9
2.4.1Δ*oxyR* -Fe	4.3 ± 0.1	21.8 ± 2.2

Doubling times were calculated for exponentially grown *R. sphaeroides* cultures in the presence and absence (-Fe) of iron in oxic or anoxic conditions. Doubling times are presented in hours (h) and represent the mean of at least three independent growth experiments.

**Figure 3:**
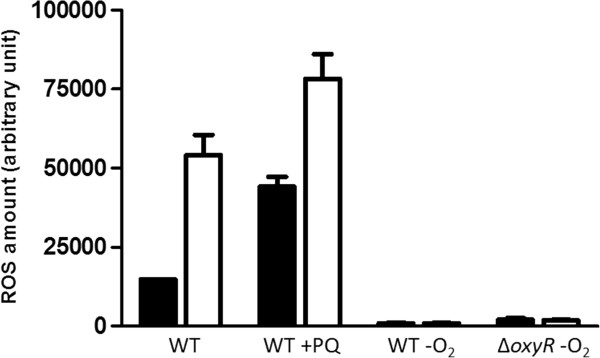
**Determination of intracellular levels of ROS in wild type** ***R. sphaeroides*** **and the 2.4.1∆** ***oxyR*** **mutant.** Cultures were grown under normal iron (black) and iron-limiting (white) conditions in oxic and anoxic (-O_2_) environments. ROS generated by the cells were analysed after reaction with 10 mM 2,7-DCFH-DA. Cells incubated with 250 μM Paraquat (PQ) served as a positive control. The autofluorescence of cells without dye was subtracted from the measured values. The fluorescence intensity was normalised to the optical densities of the samples. The resulting values are presented in arbitrary units. The data represent the mean of three independent experiments, and the error bars indicate the standard deviation.

Because some proteins involved in the oxidative stress response require iron, iron limitation also interferes with the oxidative stress defence. We therefore monitored ROS levels at different growth stages by fluorescence measurements taken after adding 10 mM 2,7-dihydrodichlorofluorescein diacetate (DCFH-DA) to the cultures. The presence of such ROS as H_2_O_2_ or hydroxyl radicals leads to elevated levels of relative DCFH-DA fluorescence (e.g., [15]). As a positive control, the wild type was stressed with the superoxide-generating paraquat for 3 hours at a final concentration of 250 μM, resulting in a 3-fold increase in ROS levels compared with the non-stressed control cultures. As previously described, exposure to oxygen and to iron starvation caused a strongly increased ROS accumulation in the wild type cells (Figure 3) [22]. The transcriptional regulator OxyR is a known key regulator of the response to hydrogen peroxide and induces the expression of antioxidant activities [39]. In the presence of iron, ROS levels in the *oxyR* deletion strain were only slightly elevated compared to those in the wild type strain at late growth stage. Under iron limitation, a clear increase in ROS levels in the *oxyR* deletion strain was observed at all tested growth stages.

Our data suggest that the elevated ROS levels under iron limitation in oxic conditions (Figure 2B) increased the level of oxidised OxyR and consequently led to the activation of OxyR-dependent genes. The increased *katE* level under oxic iron limiting conditions (log_2_ fold change 1.2, Table 1) is in agreement with this assumption. *katE* activation under oxidative stress strictly depends on OxyR.

Furthermore, a strain lacking the *oxyR* gene showed significantly reduced doubling times and a lower end-OD than the parental strain in anoxic conditions irrespective of iron availability (Table 2, Additional file 3: Figure S2). This result is in agreement with our transcript level analysis, where OxyR has nearly no notable influence on the anoxic response to iron limitation (Table 1). The possibility that the growth phenotypes were due to toxicity of the alternative electron acceptor (DMSO) can be excluded, as cultures grown in oxic conditions with DMSO did not exhibit impaired growth (data not shown). Up to now, we cannot ascribe a role to OxyR in an anoxic environment. Nevertheless, our observations indicate an important role for OxyR, even in the absence of molecular oxygen or ROS.

## Conclusion

Iron limitation increases ROS levels in *R. sphaeroides* ([22] and this study). To discriminate the effects on gene expression that are a direct consequence of iron limitation from the effects caused by the increased ROS levels under iron limitation, we compared gene expression under iron limiting conditions in the presence and absence of oxygen in *R. sphaeroides*. Of all the genes responding to iron limitation, almost one third showed the same response in the presence and absence of oxygen, implying that these genes exhibited an iron-specific response. Apparently, a link between iron availability and oxidative stress exists only for a subset of iron-dependently regulated genes.

The other two thirds of the iron-regulated genes showed diverse expression patterns that were influenced by oxygen availability, and the known regulator OxyR was identified as an important factor for induction of many genes involved in iron metabolism under oxic conditions. For those genes, elevated ROS levels were the trigger of the response rather than iron limitation itself. The OxyR regulator provides a link between the responses to oxidative stress and iron. In the future, additional regulators that are responsible for OxyR-independent activation in oxic conditions, activation in anoxic conditions and repression in response to iron limitation will need to be identified.

## Methods

### Bacterial strains and growth conditions

The strains and plasmids used in this study are listed in Additional file 4: Table S3. *E. coli* strains were grown in Luria–Bertani medium at 37°C with shaking (180 rpm) or on solid growth medium, which contained 1.6% (w/v) agar. *R. sphaeroides* strains were cultivated at 32°C in 50-ml Erlenmeyer flasks containing 40 ml malate minimal medium (Additional file 4: Table S4) with continuous shaking at 140 rpm, resulting in a constant dissolved oxygen concentration of approximately 25–30 μM during the exponential phase. These growth conditions are designated as oxic growth. To achieve anoxic conditions, we used completely filled screw-cap Meplat bottles for liquid cultures, which were sealed with Parafilm and cultivated in the dark. The remaining oxygen was used up by the cultures within 60 seconds, as confirmed using an oxygen sensor. To allow anaerobic respiration, dimethyl sulphoxide (DMSO) was added as electron acceptor at a final concentration of 60 mM. Anoxic incubation over several days resulted in a final OD_660_ of approximately 0.5. Conditions of iron limitation were achieved by transferring *R. sphaeroides* into iron-limited malate minimal medium containing the iron chelator 2,2′-dipyridyl (30 μM; Merck KGaA) three times. Inductively coupled plasma mass spectrometry (ICP-MS) using an Agilent 7500ce spectrometer confirmed that the iron content was drastically reduced in iron-limited medium (from 140 mg l^-1^ to 16 mg l^-1^) [22]. When required, antibiotics were added to liquid or solid growth media at the following concentrations: spectinomycin (10 μg ml^-1^); kanamycin (25 μg ml^-1^); tetracycline (2 μg ml^-1^) (for *R. sphaeroides*); kanamycin (25 μg ml^-1^); and tetracycline (20 μg ml^-1^) (for *E. coli*).

### Fluorescence measurements

Reactive oxygen species (ROS) generation was measured using an oxidation-sensitive fluorescent probe, 2,7-dihydrodichlorofluorescein diacetate (DCFH-DA; Molecular Probes). Cells were incubated with the probe at a final concentration of 10 μM for 30 min. The fluorescence intensities (excitation 492 nm, emission 525 nm) were evaluated in an Infiniti M200 microplate reader (Tecan).

### RNA isolation and quality assignment

*R. sphaeroides* was grown in presence or absence of external iron in triplicate cultures inoculated separately from three independent starter cultures. Cell samples were rapidly cooled on ice and harvested by cooled centrifugation. Total RNA from exponentially growing cultures with an OD_660_ of 0.5 (oxic conditions) or 0.25 (anoxic conditions) was isolated using the hot phenol method, followed by two chloroform/isoamyl alcohol treatments and precipitation with sodium acetate and ethanol. For quantitative real-time RT-PCR, RNA was isolated using the peqGOLD TriFast™ Kit (Peqlab) as described by the manufacturer. Hereupon, RNA was treated with DNase I (Invitrogen) to remove contaminating DNA. After DNA digestion, RNA was either purified by standard procedures using a mixture of phenol/chloroform/isoamyl alcohol and chloroform/isoamyl alcohol (for RT-PCR) or RNeasy® MinElute™ spin columns (Qiagen) (for microarray and RNAseq). RNA was resolved in RNase free water (Roth), and concentrations were determined using a NanoDrop 1000 Spectrophotometer (Peqlab). The absence of genomic DNA contamination was assessed by PCR using primers targeting *gloB* (RSP_0799) as described previously [40]. Polyacrylamide gels (10%, v/v) containing 7 M urea were prepared to assess RNA quality.

### Quantitative real-time RT-PCR

The One-Step Brilliant III QRT-PCR Master Mix Kit (Agilent) was used for reverse transcription followed by PCR as described in the manufacturer’s manual. RT-PCR samples containing 4 ng of total RNA per μl were run in a Rotor-Gene 3000 real-time PCR cycler (Corbett Research) for relative quantification of mRNAs in each of three independent experiments. The oligodeoxynucleotide sequences used for amplification are listed in Additional file 4: Table S5. Crossing points (Cp) with a fluorescence threshold of 0.002 were visualised with the Rotor-Gene software 6.0 (Corbett Research). Statistical comparisons were performed using the student‘s *t* test and p-values ≤0.05 were considered statistically significant. The expression of target genes was calculated relative to the control under normal iron conditions and normalised to control gene *rpoZ* [41].

### Microarray analysis

Microarray analysis was performed as described before [22]. In brief, 2 μg of total RNA of three independent experiments of control and iron-limiting cultures was pooled, chemically labelled either with Cy3 or Cy5 and hybridised to one array. Transcriptome profiles were analysed on two arrays including six biological replicates. Differentially labelled RNA samples were mixed and competitively hybridised to microarrays. Hybridisations and scanning were performed according to the specifications from Agilent. Multiarray analysis and normalisation according to LOESS were accomplished with the Bioconductor package Limma for R and performed as described elsewhere [42, 43]. On the basis of calculated MA plots, genes were considered reliable if the average signal intensity [A-value: 1/2 log2 (Cy3 × Cy5)] was ≥ 12. Fold changes were calculated using MS Excel (Microsoft). The data shown in this study represent the results from two individual microarrays (biological replicates), each containing a pool of three independent experiments for each sample. The microarray data have been deposited in NCBI’s Gene Expression Omnibus [44] and are accessible through GEO Series accession number GSE47182 (http://www.ncbi.nlm.nih.gov/geo/query/acc.cgi?acc=GSE47182).

### RNA-sequencing

#### Library construction and sequencing

The libraries were generated by Vertis Biotechnologie AG (Munich, Germany). The treated and untreated RNA samples were poly(A)-tailed by using poly(A) polymerase. The 5′-PPPs were removed using tobacco acid pyrophosphatase (TAP) followed by the ligation of the RNA adapter to the 5′-monophosphate of the RNA. First-strand cDNA synthesis was performed with an oligo(dT)-adapter primer and M-MLV reverse transcriptase. The resulting cDNA was PCR-amplified to reach a concentration of 20–30 ng/μl using a high fidelity DNA polymerase. The cDNA was purified using the Agencourt AMPure XP kit (Beckman Coulter Genomics) and was analysed by capillary electrophoresis.

The primers used for PCR amplification were designed for TruSeq sequencing according to the recommendations of Illumina. The following adapter sequences flank the cDNA inserts: TrueSeq_Sense_primer 5′-AAT GAT ACG GCG ACC GAG ATC TAC ACT CTT TCC CTA CAC GAC GCT CTT CCG ATC T-3′; TrueSeq_Antisense_NNNNNN_primer (NNNNNN = Barcode) 5′-CAA GCA GAA GAC GGC ATA CGA GAT-NNNNNN-GTG ACT GGA GTT CAG ACG TGT GCT CTT CCG ATC (dT25)-3′. The combined length of the flanking sequences is 146 bases. The libraries were sequenced with an Illumina HiSeq machine with 100 cycles in single-end mode. The data shown in this study represent the results from one RNAseq experiment containing a pool of three independent experiments for each sample.

### Bioinformatic analyses

The pooled sequence reads from the RNAseq were demultiplexed, and the adapter sequences were removed. After that, the reads in Fastq format were quality trimmed using *fastq_quality_trimmer* (from the FastX suite version 0.0.13 - http://hannonlab.cshl.edu/fastx_toolkit/) with a cut-off Phred score of 20 and converted to fasta format using *fastq_to_fasta* (also from the FastX suite). The read processing (poly(A) removal, size filtering (min 12 nt length), statistics generation, gene-wise read counting, RPKM and coverage calculation as well as normalisation was performed using READemption [24] with default parameters using *segemehl* version 0.1.3 [25] for the read alignment (accuracy parameter set to 95%). Differential gene expression analysis was performed with DESeq 1.12.0 [26] only considering genes that show in at least one the conditions a RPKM [27] value of ≥ 5.0.

The sequences and annotations of the replicons with the following GenBank Ids were used as references: CP000143.1, CP000144.1, CP000145.1, CP000146.1, CP000147.1, DQ232586.1, DQ232587.1 (Additional file 5: Table S6). The demultiplexed Fastq files and coverage files in wiggle format have been deposited in NCBI’s Gene Expression Omnibus [44] and are accessible through GEO Series accession number GSE47182 (http://www.ncbi.nlm.nih.gov/geo/query/acc.cgi?acc=GSE47182).

Cluster analysis was performed using MeV (Multi Experiment Viewer version 4.7.4) from the TM4 Microarray Software Suite [44–46]. Clustering was based on k-means (KMC method) according to Euclidean distance with a maximum of 50 iterations. Strongly regulated genes (log_2_ ratio > 1 or < -1) were grouped into six clusters and visualised as a scatter-plot (Figure 1B).

### Ethical approval

No ethical approval was required in connection with the study as there was no research involving human subjects or vertebrates.

## Availability of supporting data

All data sets supporting the results of this article are accessible in the NCBI’s GEO database through GEO Series accession number GSE47182 (http://www.ncbi.nlm.nih.gov/geo/query/acc.cgi?acc=GSE47182).

## Electronic supplementary material

Additional file 1: Table S1: Gene expression changes under iron limitation as determined by microarray analysis and RNAseq. (XLSX 984 KB)

Additional file 2: Table S2: Gene expression changes under iron limitation as determined by RNAseq. All strongly regulated genes were grouped into six clusters based on the different expression profiles, compared to real-time RT-PCR analysis with corresponding p-values. (XLSX 339 KB)

Additional file 3: Figure S1: Log_2_ fold changes in response to iron limitation in oxic conditions and presence of DMSO (60 mM) as determined by real-time RT-PCR. Numbers in parentheses show the log_2_ fold change of the respective genes as determined by real-time RT-PCR in absence of DMSO (Table 1). Values are normalised to *rpoZ* and to the control under normal iron conditions. The data represent the mean of at least three independent experiments and error bars indicate standard deviation. **Figure S2.** Growth curves of wild type *R. sphaeroides* (black) and the 2.4.1∆*oxyR* mutant (grey) in anoxic conditions in the presence (continuous line) or absence (dashed line) of iron are shown. The optical density at 660 nm (OD_660_) was determined over time. The data represent the mean of at least three independent experiments. (PDF 113 KB)

Additional file 4: Table S3: Bacterial strains and plasmids. **Table S4.** Composition of malate minimal medium. **Table S5.** Oligodeoxynucleotide sequences and primer efficiencies for real-time RT-PCR. (PDF 588 KB)

Additional file 5: Table S6: Mapping of RNA sequencing reads to the genome of *R. sphaeroides*. (XLSX 11 KB)
