# Supplementary figures and images for: Role of oxygen and the OxyR protein in the response to iron limitation in Rhodobacter sphaeroides
Source: BMC Genomics. 2014 Sep 15;15(1):794. doi: 10.1186/1471-2164-15-794 (PMC4176601; doi:10.1186/1471-2164-15-794)

Figure S1

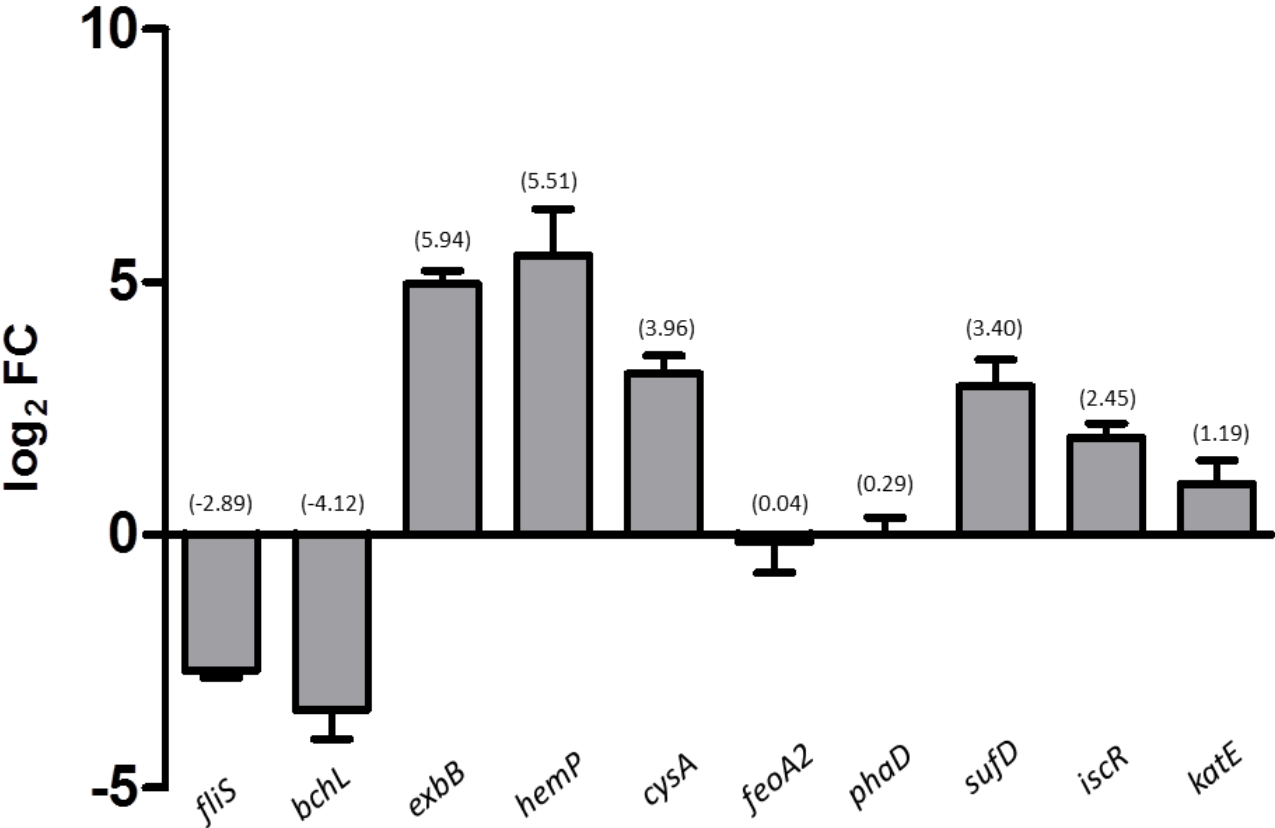

Figure S2

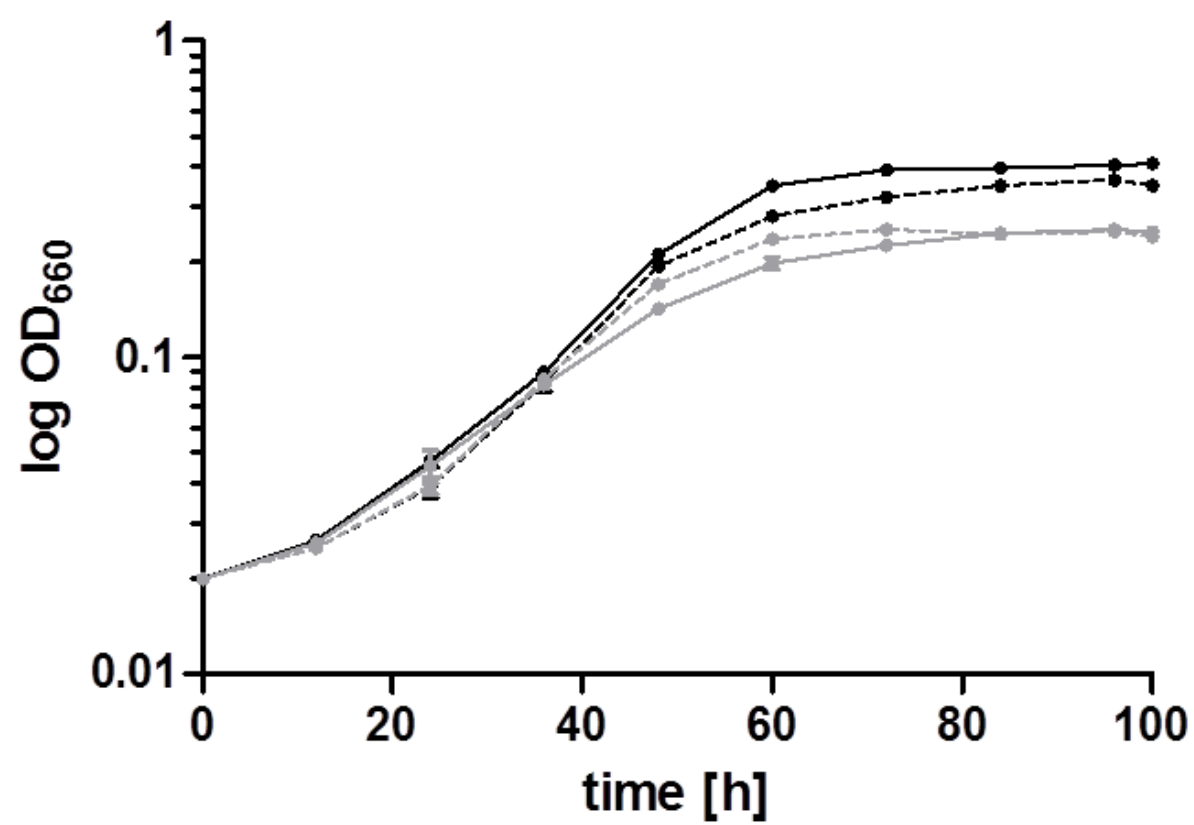

Supplement: Supplementary file 3 — Additional file 3: Figure S1: Log2 fold changes in response to iron limitation in oxic conditions and presence of DMSO (60 mM) as determined by real-time RT-PCR. Numbers in parentheses show the log2 fold change of the respective genes as determined by real-time RT-PCR in absence of DMSO (Table 1). Values are normalised to rpoZ and to the control under normal iron conditions. The data represent the mean of at least three independent experiments and error bars indicate standard deviation. Figure S2. Growth curves of wild type R. sphaeroides (black) and the 2.4.1∆oxyR mutant (grey) in anoxic conditions in the presence (continuous line) or absence (dashed line) of iron are shown. The optical density at 660 nm (OD660) was determined over time. The data represent the mean of at least three independent experiments. (PDF 113 KB) [file 12864_2014_6469_MOESM3_ESM.pdf]
