# Supplementary material for: Role of oxygen and the OxyR protein in the response to iron limitation in Rhodobacter sphaeroides
Source: BMC Genomics. 2014 Sep 15;15(1):794. doi: 10.1186/1471-2164-15-794 (PMC4176601; doi:10.1186/1471-2164-15-794)
Supplement: Supplementary file 4 — Additional file 4: Table S3: Bacterial strains and plasmids. Table S4. Composition of malate minimal medium. Table S5. Oligodeoxynucleotide sequences and primer efficiencies for real-time RT-PCR. (PDF 588 KB) [file 12864_2014_6469_MOESM4_ESM.pdf]

Additional file 4

Table S3. Bacterial strains and plasmids

Strain	Description	Reference or source
***R. sphaeroides* strains**		
2.4.1	Wild-type	[1]
2.4.1Δ*oxyR*	2.4.1Δ*oxyR*:Sp^r	[2]
2.4.1Δ0019	2.4.1Δ0019:Km^r	this study
***E. coli* strains**		
JM109	Cloning strain	New England Biolabs
S17-1	Donor strain for diparental conjugation	[3]
**Plasmids**		
pPHU281	Tc^r, *lacZ' mob*(RP4)	[4]
pUC4K	source of kanamycin cassette	[5]
pRK415 Tc^r	broad-host-range cloning vector	[6]
pPHU0019::$Km^{r\wedge}$	fragment containing the upstream and downstream regions of the *R. sphaeroides* IGR0019 and the kanamycin cassette cloned into pPHU218	this study

Sp^r, spectinomycin-resistant; Km^r, kanamycin-resistant; Tc^r, tetracycline-resistant

Table S4. Composition of malate minimal medium

Compound	mg/l	Final concentration in the growth medium
Malic acid	3000	22 mM
$MgSO_4 \cdot 7H_2O$	200	0.8 mM
NH_4Cl	1200	22 mM
$CaCL_2 \cdot 2H_2O$	70	0.5 mM
K_2HPO_4	900	5 mM
KH_2HPO_4	600	4.4 mM
Fe(II)-Citrat	0.75	3 µM
$MnCl_2 \cdot 4H_2O$	0.03	150 nM
$ZnCl_2$	0.007	50 nM
LiCl	0.007	150 nM
KJ	0.0035	20 nM
$CuSO_4$	0.00025	1.5 nM
$Na_2MoO_4 \cdot 2H_2O$	0.0015	6 nM
$CoCl_2 \cdot 6H_2O$	0.0075	30 nM
$SnCl_2 \cdot 2H_2O$	0.00075	3 nM
$BaCl_2$	0.00075	3 nM
$AlCl_3$	0.0015	12 µM
H_3BO_4	0.015	1.5 nM
EDTA	0.03	100 nM
Thiamin	3.2	10 µM
Niacin	1.6	13 µM
Nicotinamid	1.6	13 µM
Biotin	0.064	0.3 µM
Water (pH 6.9)	1 L	

Table S5. Oligodeoxynucleotides and primer efficiencies used for real-time RT-PCR.

Corresponding gene	Primer	Sequence 5`-3`	Efficiency	Reference
RSP_0069 (*fliC*)	A	TCGAACGACACCAACACC	1.92	this study
	B	CACCTTCATGCCGTTGAA		
RSP_0130 (*metI*)	A	CACGCTTCAGACGCTCTACA	2.01	this study
	B	GAATAGCCGATGAGGCTGAC		
RSP_0261 (*bchY*)	A	CAATTCGGAATCGCTCGT	1.99	[7]
	B	ACGCAGAGGTTCGTCACC		
RSP_0285 (*bchN*)	A	TCGAGGAGAAGGATCTCG	2.19	[7]
	B	GAGCCGGAGAAGTTGTAGAC		
RSP_0288 (*bchL*)	A	CGATCAGGCGGTGGTGGT	2.16	this study
	B	CGTCGAGGTCCGGCATGT		
RSP_0434 (*sufD*)	A	CGCGGTTCTGCACCATTG	1.97	[8]
	B	TTGGCCGTCAGCGTGAAG		
RSP_0437 (*sufC*)	A	GAGAAGAAGCGCAACGCG	2.03	this study
	B	GCGGATATGATCGACCAG		
RSP_0439	A	CAACGTGATCCGCGACCT	2.11	this study
	B	GGCCGACACCATCAGCAT		
RSP_0440 (*sufB*)	A	ACATCGTGCGGCTGATCT	2.11	[8]
	B	CGGCTTCACATCCATGCT		
RSP_0443 (*iscR*)	A	GGTGGAAGAGACGCTCAA	2.15	this study
	B	ATAGACATGCGCCGAGAC		
RSP_0601 (*rpoH$_{II}$*)	A	GCCGATGAACGACCTGAT	2.09	[9]
	B	AAGAACAGCGCCTTCTGG		
RSP_0679 (*hemC*)	A	GCGGTCCATTCGATGAAG	1.99	[10]
	B	TCGGCGAAGGTGATGAAG		
RSP_0850 (*mbfA*)	A	GCGTCGGCAGTTCATCCT	2.09	[8]
	B	GAGAGCACGCCATCGTCA		
RSP_0906 (*sitC*)	A	TCAAGACCGACGTGGTAA	2.06	this study
	B	AGCAGCGTGAAGAACAGA		
RSP_0920 (*exbB*)	A	GCGAGCTTCGAGGAACTG	2.01	[11]
	B	GTCTGGCTCTGCGAGATG		
RSP_0921 (*exbD*)	A	GATCCTCGTGCTGCTCAT	2.08	this study
	B	CGACAGCGTCAGATCCTT		
RSP_0922 (*tonB*)	A	TGCAGGAGGCGCTCTATC	1.88	this study
	B	CGGCCACCTCTTCCTCTT		
RSP_0994 (*phaD*)	A	GTCTTCTTCGAGGTTCTG	2.02	[12]
	B	GGTTGTAGGCCACATACT		
RSP_1092 (*rpoE*)	A	GTCTGGCAGAAGGCTCAT	2.04	[9]
	B	GTTCTCCTGCTGCATCTC		
RSP_1109 (*cysK*)	A	ACGTCAAGGCCGAGTTCT	1.92	this study
	B	GAAGGCTTCGGACATGGT		
RSP_1112 (PnP)	A	GACTATGCCGCGCTCTAT	2.09	[12]
	B	GCTCGTCTCCGAGATGAT		
RSP_1438	A	CGTCCTCTCCACCATCTT	1.95	this study
	B	GACGAGCGACAGCAGATA		
RSP_1547 (*bfd*)	A	GTCTGCCACTGCATGGGCAT	1.81	[11]
	B	GTTCATTCCTGCGGTTGTGG		
RSP_1548 (*irpA*)	A	ATGGGCAGCCTCTCCTAC	2.34	[11]
	B	GTGTTGTCCGAGAAACAGTC		
RSP_1565 (*appA*)	A	CACCAACATCTTCTCTTTCG	1.94	[8]
	B	CATAAAGGGCGAAGGTGT		
RSP_1669 (*rpoZ*)	A	ATCGCGGAAGAGACCCAGAG	2.02	[13]
	B	GAGCAGCGCCATCTGATCCT		
RSP_1818 (*feoA1*)	A	CCGCCAGAAGCTGCTCTC	2.03	this study
	B	CGCAGCGACAGCTGGTAG		
RSP_2311 (*groEL*)	A	GTCATCACCGTCGAAGAG	2.01	[9]
	B	TCGTGGAGCAGGATGTAG		
RSP_2395 (*ccpA*)	A	TCGAGGCGACGCTCATCA	2.08	[8]
	B	TCGATCAGGCCGAACGGA		
RSP_2779 (*katE*)	A	CTATCCGCTGATCGAGGT	2.07	[2]
	B	GTCGGCATAGGAGAAGAC		
RSP_2913 (*afuA*)	A	CATCAGCCTCGGCAACAC	2.15	[11]
	B	TGGTTCGTCTCGGCGTAG		

RSP_3323	A	GTGATCGGCTCGAACAAC	2.00	this study
	B	GCGCTGGATCTCCTCATA		
RSP_3567 (*znuB*)	A	CTGGCTCTTCGGCGACAT	1.81	this study
	B	GCGGCCAGATCCTCATTG		
RSP_3568 (*znuC*)	A	CCGGCTGATCGAAGAGGT	1.86	this study
	B	GCAGACATGGCGGTTGAG		
RSP_3696 (*cysA*)	A	AAGGTAGCCGACAACGTG	2.01	this study
	B	CTCGTCGAGAAGCAGGAT		
RSP_3697 (*cysP*)	A	AATGCGCGCTACACCTATCT	2.00	this study
	B	GTCATGCACCCGGTAGAAAT		
RSP_6006 (*hemP*)	A	ATGACCGACCCGATGGAG	2.05	[11]
	B	AGTAGATCTGCCCGTCGAG		
RSP_6020 (*feoA2*)	A	CGGCGAGGTCAAGGTGAT	2.19	this study
	B	TGCAGCGGAGTGACGAAG		
RSP_6158 (*puc2A*)	A	GGCAAAATCTGGCTCGTGGT	2.05	(7)
	B	GGTGGTGGTCGTCAGCACAG		

1. van Niel CB: **The Culture, General Physiology, Morphology, and Classification of the Non-Sulfur Purple and Brwon Bacteria**. *Bacteriol Rev* 1944, **8**:1-118.
2. Zeller T, Klug G: **Detoxification of hydrogen peroxide and expression of catalase genes in Rhodobacter**. *Microbiology* 2004, **150**(Pt 10):3451-3462.
3. Simon R, O'Connell M, Labes M, Puhler A: **Plasmid vectors for the genetic analysis and manipulation of rhizobia and other gram-negative bacteria**. *Methods Enzymol* 1986, **118**:640-659.
4. Hubner P, Willison JC, Vignais PM, Bickle TA: **Expression of regulatory nif genes in *Rhodobacter capsulatus***. *J Bacteriol* 1991, **173**(9):2993-2999.
5. Vieira J, Messing J: **The pUC plasmids, an M13mp7-derived system for insertion mutagenesis and sequencing with synthetic universal primers**. *Gene* 1982, **19**(3):259-268.
6. Keen NT, Tamaki S, Kobayashi D, Trollinger D: **Improved broad-host-range plasmids for DNA cloning in gram-negative bacteria**. *Gene* 1988, **70**(1):191-197.
7. Mank NN, Berghoff BA, Hermanns YN, Klug G: **Regulation of bacterial photosynthesis genes by the small noncoding RNA PcrZ**. *Proceedings of the National Academy of Sciences of the United States of America* 2012, **109**(40):16306-16311.
8. Peuser V, Remes B, Klug G: **Role of the Irr protein in the regulation of iron metabolism in Rhodobacter sphaeroides**. *PloS one* 2012, **7**(8):e42231.
9. Nuss AM, Glaeser J, Klug G: **RpoH(II) activates oxidative-stress defense systems and is controlled by RpoE in the singlet oxygen-dependent response in Rhodobacter sphaeroides**. *Journal of bacteriology* 2009, **191**(1):220-230.
10. Metz S, Jager A, Klug G: **Role of a short light, oxygen, voltage (LOV) domain protein in blue light- and singlet oxygen-dependent gene regulation in Rhodobacter sphaeroides**. *Microbiology* 2012, **158**(Pt 2):368-379.
11. Peuser V, Metz S, Klug G: **Response of the photosynthetic bacterium *Rhodobacter sphaeroides* to iron limitation and the role of a Fur orthologue in this response**. *Environmental Microbiology Reports* 2011, **3**(3):397-404.
12. Fruhwirth S, Teich K, Klug G: **Effects of the cryptochrome CryB from Rhodobacter sphaeroides on global gene expression in the dark or blue light or in the presence of singlet oxygen**. *PloS one* 2012, **7**(4):e33791.
13. Gomelsky L, Sram J, Moskvin OV, Horne IM, Dodd HN, Pemberton JM, McEwan AG, Kaplan S, Gomelsky M: **Identification and in vivo characterization of PpaA, a regulator of photosystem formation in Rhodobacter sphaeroides**. *Microbiology* 2003, **149**(Pt 2):377-388.
